# Investigation of tolerability and quality of life for carboplatin-based chemotherapy in an elderly urothelial carcinoma patient undergoing hemodialysis: a case report

**DOI:** 10.1186/s40780-018-0124-0

**Published:** 2018-11-29

**Authors:** Masahiro Kondo, Taku Naiki, Yuji Hotta, Yuko Yamamoto, Yosuke Sugiyama, Takahiro Yasui, Kazunori Kimura

**Affiliations:** 1 Department of Pharmacy, Nagoya City University Hospital, 1-Kawasumi, Mizuho-cho, Mizuho-ku, Nagoya, Aichi 467-8602 Japan; 2 Department of Nephro-urology, Nagoya City University Graduate School of Medical Sciences, 1-Kawasumi, Mizuho-cho, Mizuho-ku, Nagoya, Aichi 467-8602 Japan; 3 Department of Hospital Pharmacy, Graduate School of Pharmaceutical Sciences, Nagoya City University, 3-1, Tanabe dori, Mizuho ku, Nagoya, Aichi 467-8603 Japan; 4 Aichi Prefectural Institute of Public Health, 7-6, Tsuji machi, Kita ku, Nagoya, Aichi 462-8576 Japan; 5 Department of Clinical Pharmaceutics, Nagoya City University Graduate School of Medical Sciences, 1-Kawasumi, Mizuho-cho, Mizuho-ku, Nagoya, Aichi 467-8602 Japan

**Keywords:** Carboplatin, Hemodialysis, Pharmacokinetics, Quality of life, Elderly

## Abstract

**Background:**

To our knowledge, no studies have evaluated the safety of carboplatin (CBDCA)-based chemotherapy in hemodialysis patients > 80 years-old. In addition, the impact of CBDCA-based chemotherapy on such elderly patients’ quality of life (QOL) is unknown. We report a case of gemcitabine plus CBDCA chemotherapy treatment in an 81-year-old man with metastatic urothelial carcinoma undergoing hemodialysis.

**Case presentation:**

The optimal CBDCA dose and hemodialysis timing were determined by monitoring the measured area under the concentration–time curve (AUC) of CBDCA. This was used because the AUC of CBDCA is correlated with hematologic toxicities, especially nadir thrombocytopenia, and CBDCA is easily dialyzed during hemodialysis. In the first cycle, a 160 mg CBDCA dose, calculated using Calvert’s formula (target-AUC: 5), was administered on day 1. Hemodialysis was performed for 3 h, starting 2 h after the end of the CBDCA infusion. The measured-AUC was 5.96 mg/mL min in the first cycle, after which the patient developed grade 3/4 hematologic toxicities. Thus, in the second cycle, the CBDCA dose was reduced to 135 mg and the time interval between CBDCA infusion and hemodialysis was shortened to 1 h, according to the results of a pharmacokinetic study performed using parameters from the first cycle. The measured-AUC in the second cycle was 4.97 mg/mL min, and hematologic toxic effects decreased to grade 2. Stable disease according to the Response Evaluation Criteria in Solid Tumors was demonstrated after the second and third cycles. QOL scores determined using a short-form questionnaire (SF-36) after 2 cycles were not significantly lower than pretreatment values.

**Conclusions:**

CBDCA-based chemotherapy is clinically acceptable in hemodialysis patients aged > 80 years, and this systemic chemotherapy can be a treatment option in such elderly patients undergoing hemodialysis. However, the measured-AUC should be monitored, as the actual AUC is unpredictable in hemodialysis patients. This is due to the influence of various factors that may be different for each patient, such as the patient’s residual renal function and hemodialysis duration and conditions, especially in elderly patients, who have a higher risk of chemotherapy-induced neutropenia.

## Background

The National Comprehensive Cancer Network clinical practice guidelines recommend systemic cisplatin-based chemotherapy in patients with urothelial carcinoma (UC) who have metastatic or recurrent disease following radical surgery [1]; the gemcitabine (GEM) plus cisplatin regimen is commonly used in clinical practice [1, 2]. However, because cisplatin is highly protein bound, its acute and chronic toxicity profile is unpredictable in patients undergoing hemodialysis. Therefore, carboplatin (CBDCA), a second-generation platinum agent, is preferred in patients with renal failure because of its predictable kinetics and limited toxicity profile [3]. Some reports have described the use of CBDCA-based chemotherapy in cancer patients undergoing hemodialysis; however, these patients were < 80 years old [4–9].

For the management of side effects associated with CBDCA, it is important to control the area under the concentration–time curve (AUC) of CBDCA after intravenous administration, because the AUC of CBDCA is correlated with hematologic toxicities, especially nadir thrombocytopenia [10]. Thus, the CBDCA dose should be calculated using Calvert’s formula, which incorporates the pretreatment glomerular filtration rates (GFR) along with the AUC of CBDCA [10]. An AUC of 5.0 mg/mL min is commonly recommended for patients with locally advanced or metastatic UC [11]. With regards to the administration of CBDCA for hemodialysis patients, prior reports describe the importance of the setting of the CBDCA dose or removal by hemodialysis to control the AUC [4, 9]. However, standard procedures have not yet been established for CBDCA administration to hemodialysis patients because the administration of anticancer agents to such patients, especially in elderly individuals, is very rare. In addition, no studies have evaluated the quality of life (QOL) of hemodialysis patients receiving CBDCA-based chemotherapy.

We describe the case of an elderly hemodialysis patient with UC who was treated with GEM plus CBDCA chemotherapy by monitoring the measured AUC of CBDCA to determine the optimal dose and hemodialysis timing. Moreover, we also report the results of a QOL survey administered while the patient was receiving chemotherapy.

## Case presentation

The patient was an 81-year-old Japanese man. He was diagnosed with a recurrence of UC with multiple lymph node metastases, originating from the left renal pelvis, 1 year after laparoscopic radical nephroureterectomy. In addition, his renal function worsened 1 month before the above diagnosis; a shunt was created surgically for hemodialysis initiation. Thus, chemotherapy was planned while the patient continued hemodialysis.

Before the commencement of chemotherapy, the patient’s Eastern Cooperative Oncology Group performance status was 1. His height was 163.5 cm, dry weight was 51.90 kg, and body surface area (BSA) was 1.55 m^2^. Laboratory findings were as follows: white blood count, 3700/μL; hemoglobin, 11.0 g/dL; platelet count, 168 × 10^3^/μL; blood urea nitrogen, 24.2 mg/dL; and creatinine, 3.38 mg/dL. The patient had residual renal function, with daily urine volume > 500 mL and a 24-h creatinine clearance of 7.3 mL/min.

The GEM dose was reduced by 25% (750 mg/m^2^) and was administered by intravenous infusion for 30 min on days 1 and 8 of a 21-day cycle. CBDCA was administered by intravenous infusion for 60 min on day 1, followed by infusion of GEM. The initial CBDCA dose was calculated according to the Calvert’s formula (target AUC: 5.0 mg/ml min, GFR: 6.1 mL/min). GFR was calculated based on knowledge that 24-h creatinine clearance is generally approximately 20% higher than GFR [12]. Therefore, the CBDCA dose was calculated as 160 mg.

Hemodialysis commenced 2 h after the end of CBDCA infusion on day 1 and was performed for 3 h, with a blood flow rate of 200 mL/min and a continuous infusion of heparin as an anticoagulant. The dialyzer membrane was made of polymethyl methacrylate, with a surface area of 1.6 m^2^ (BK1.6P, TORAY Inc., Tokyo, Japan). Subsequent hemodialysis was performed on days 3 and 5.

**Fig. 1:**
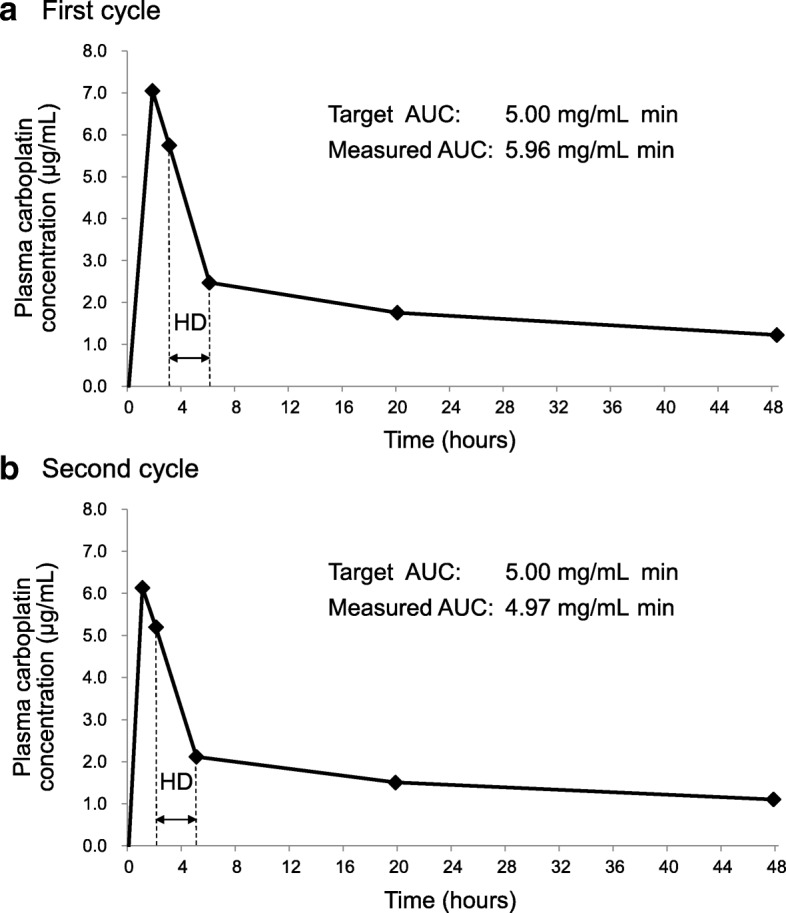
Plasma concentration–time curves of carboplatin during the (**a**) first and (**b**) second chemotherapy cycle. The time intervals between the carboplatin infusion and the commencement of hemodialysis were 2 h in the first cycle and 1 h in the second cycle of gemcitabine plus carboplatin chemotherapy. Hemodialysis was performed for 3 h in each chemotherapy cycle. AUC: area under the concentration-time curve; HD, hemodialysis

A pharmacokinetic study was performed to monitor the measured AUC of CBDCA. This study was approved by the ethical review board at Nagoya City University Graduate School of Medical Sciences. Informed consent was obtained from the patient. Blood samples were collected during the first 2 cycles of chemotherapy. Sampling points were as follows: immediately after CBDCA infusion, before starting and ending hemodialysis, and 20 and 48 h after CBDCA infusion on day 1 (Fig. 1). The plasma was stored at − 80 °C until analysis. The plasma platinum level was measured by inductively coupled plasma mass spectrometry. The CBDCA level was calculated using the molar ratio of platinum: CBDCA (371.25/195.08). The measured AUC of plasma CBDCA was calculated using the trapezoidal method according to the intervals before, during, and after hemodialysis, with extrapolation to infinity.

The measured AUC of CBDCA in the first cycle was 5.96 mg/mL min, which was 19.2% higher than the target AUC (Fig. 1a). Consequently, grade 4 neutropenia and grade 3 thrombocytopenia were observed, according to the National Cancer Institute’s Common Toxicity Criteria for Adverse Events version 4.0 (Table 1). To match the target AUC more closely in the subsequent cycle, we performed a pharmacokinetic study using parameters obtained in the first cycle. Consequently, the CBDCA dose was reduced to 135 mg, and the time interval between the CBDCA infusion and hemodialysis initiation was shortened to 1 h in the second cycle. The hemodialysis duration and conditions, such as the dialyzer and blood or dialysate flow rates, were unchanged. The measured AUC of CBDCA in the second cycle was 4.97 mg/mL min (Fig. 1b). The CBDCA dose and administration procedure in the third cycle were the same as that in the second cycle. In the second and third cycles, neutropenia severities were grades 2 and 3 and thrombocytopenia severities were grades 2 and 1, respectively (Table 1). In addition, no other serious adverse events, including nausea and vomiting, were observed by the supportive care. Serum creatinine levels immediately prior to starting the second and third cycles were 3.12 mg/dL and 2.84 mg/dL, respectively; the 24-h creatinine clearance was only measured prior to the first cycle. The CBDCA removal rates by hemodialysis in the first and second cycles were calculated at 56.9 and 59.3%, respectively, though the redistribution phenomenon in the post hemodialysis period could not be considered due to the small number of blood sampling points. Other pharmacokinetic parameters in the first and second cycles are shown in Table 2. Total clearance of CBDCA was slightly higher in the second cycle than in the first cycle. Stable disease (according to Response Evaluation Criteria in Solid Tumors) was demonstrated by computed tomography after the second and third cycles.

**Table 1:** The measured AUC of carboplatin and hematologic toxic effects for 3 cycles of chemotherapy

Cycle	Doses of anticancer agents (mg)		Measured AUC of CBDCA (mg/mL min)	Neutrophil count (/μL)		Platelet count (×10^3^/μL)		Hemoglobin (g/dL)	
	CBDCA	GEM		Pretreatment	Nadir	Pretreatment	Nadir	Pretreatment	Nadir
First	160	1160	5.96	2100	315	168	40	11.0	8.2
Second	135	1160	4.97	5733	1248	285	60	11.0	9.5
Third	135	1160	–	4095	800	258	129	9.5	8.5

*CBDCA* carboplatin, *GEM* gemcitabine, *AUC* area under the concentration-time curve

**Table 2:** Pharmacokinetic parameters of carboplatin in the first and second cycle of gemcitabine plus carboplatin chemotherapy

Treatment phase	First cycle				Second cycle			
	Measured AUC (mg/mL min)	Ke (h^−1^)	t_1/2_ (h)	CL (mL/min)	Measured AUC (mg/mL min)	Ke (h^−1^)	t_1/2_ (h)	CL (mL/min)
Pre-dialysis	0.88	0.150	4.6	–	0.47	0.211	3.3	–
During dialysis	0.74	0.281	2.5	–	0.66	0.300	2.3	–
Post-dialysis	4.34	0.017	40.8	–	3.84	0.015	46.2	–
Total	5.96	–	–	26.8	4.97	–	–	27.2

Carboplatin dose in the first and second cycle were 160 mg and 135 mg, respectively. The time interval between the carboplatin infusion and hemodialysis initiation in the first and second cycle were two hours and one hour, respectively

*AUC* area under the concentration–time curve of carboplatin, *Ke* elimination rate constant, *t*_*1/2*_ elimination half-life, *CL* clearance

The patient’s QOL before treatment and after 2 cycles of treatment was evaluated using the Medical Outcomes Study 36-Item Short Form Survey (SF-36, iHope International Inc., Kyoto, Japan), which is a questionnaire used to measure general health status [13]. Mean norm-based score (NBS) is an international common score recalculated on the basis of the score of 8 items of the SF-36. Our patient’s NBS did not significantly decrease after 2 cycles of chemotherapy compared with his NBS before treatment (Table 3).

**Table 3:** The change in mean NBS for QOL before and after 2 cycles of chemotherapy

Scale name	Mean NBS	
	Pre-chemotherapy	After 2 cycles
Physical functioning	25.4	29.0
Role physical	52.4	49.1
Bodily pain	66.7	66.7
General health perception	45.8	45.8
Vitality	43.4	40.2
Social functioning	57.0	57.0
Role emotional	51.9	47.7
Mental health	59.8	57.2

The patient’s QOL was evaluated using the Medical Outcomes Study 36-Item Short Form Survey (SF-36). This survey consisted of 8 items. Mean NBS is an international common score recalculated on the basis of the score on 8 items of the SF-36

*NBS* norm-based score, *QOL* quality of life

## Discussion and conclusions

No studies have investigated the safety and the impact on QOL of CBDCA-based chemotherapy in elderly hemodialysis patients > 80 years old. Herein, we describe the treatment tolerability and effects on QOL of GEM plus CBDCA chemotherapy in such a patient. The initial CBDCA dose, which was calculated using Calvert’s formula, and hemodialysis timing were determined according to prior reports. However, the measured AUC in the first cycle unexpectedly exceeded the target AUC by about 20%, resulting in the development of grade 4 hematologic toxicities. A prior report concluded that Calvert’s formula causes CBDCA overdosing by overestimating CBDCA clearance in adult patients with severe renal insufficiency, including those undergoing hemodialysis, because this formula was originally developed using pharmacokinetic data from Caucasian patients with a GFR ranging from 33 to 135 mL/min [5, 10]. This might explain why the measured AUC exceeded the target AUC in our patient, although this has not been investigated in studies with large numbers of patients.

On the other hand, in a hemodialysis patient, the CBDCA AUC is dependent upon not only its dose, but also the hemodialysis timing. According to some prior reports, hemodialysis timing can be roughly classified as commencing on the day of CBDCA administration (commencement 1 to 2 h after the end of CBDCA infusion) or commencing on the day after CBDCA administration (commencement 16 to 24 h after the end of CBDCA infusion) [4–9]. However, the correlation between these 2 hemodialysis timings and the ratio of measured AUC to target AUC has not been established. Moreover, there are also other potential factors which affect the AUC of CBDCA, including the patient’s residual renal function and hemodialysis duration and conditions [8]. Various factors in each patient may affect the AUC of CBDCA. Therefore, especially in the first administration of CBDCA, the prediction of the actual AUC following CBDCA infusion is very difficult in patients who are undergoing hemodialysis, although some reports have described that CBDCA-based chemotherapy can be administered to such patients. Therefore, in cases receiving chemotherapy with non-curative intent, similar to our hemodialysis patient, the initial dose of anti-cancer agents in the first cycle should be reduced to take into account the patient’s safety and QOL.

When chemotherapy-induced adverse events occur, especially hematologic toxicities, dose reduction is a useful method for decreasing such toxicity, because the AUC of CBDCA may be reduced in cases receiving CBDCA-based chemotherapy. However, in hemodialysis patients, it is uncertain whether the AUC of CBDCA and associated toxicities will decrease enough by only standardized dose reduction, as in non-hemodialysis patients, because the AUC of CBDCA is not dependent upon only the dose. Ineffective treatment will result if the dose is reduced too much, although there may be decreased toxicity. Without monitoring the AUC, it would be difficult to discover this insufficient treatment intensity. Therefore, monitoring of the measured AUC following the administration of CBDCA is important as the indicator for setting the appropriate dose or for hemodialysis timing in each hemodialysis patient.

It was previously reported that the total clearance of CBDCA in the second and third cycle decreased to 30.0 mL/min and 20.8 mL/min, respectively, compared to 43.7 mL/min in the first cycle, when the CBDCA dose remained unchanged, and the time interval in the second and third cycle was extended from 1 h to 16 and 20 h, respectively [4]. Thus, the extension of the time interval between CBDCA infusion and hemodialysis initiation could lead to a decrease in the total clearance of CBDCA through the treatment period and then to an increase in the AUC of CBDCA. In our case, we changed both the dose of CBDCA and the hemodialysis timing from the second cycle. The dose was reduced from 160 mg to 135 mg, and the time interval between CBDCA infusion and hemodialysis initiation was shortened by 1 h. Consequently, there was a decrease in the measured AUC of CBDCA in the second cycle by about 17%, compared that in to the first cycle. However, the increase in the total clearance in the second cycle was minor compared to that in the first cycle. This may have been due to the extended time interval between CBDCA infusion and the fact that hemodialysis initiation was only 1 h. Thus, in our case, the dose reduction might have more strongly contributed to the decrease in the AUC of CBDCA than shortening the time interval between CBDCA infusion and hemodialysis initiation. However, these results in our study do not suggest that the hemodialysis timing does not contribute to the change in AUC of CBDCA and CBDCA-related side effects, considering previous reports described above [4]. Therefore, monitoring of the measured AUC following the administration of CBDCA is needed, even if there is a short time interval between CBDCA infusion and hemodialysis.

The lack of 24-h creatinine clearance data for the second and third cycle may be a limitation of this study. Following discussion with the physician, we only measured the 24-h creatinine clearance before the first cycle, because the patient’s general condition was stable and the patient’s burden with urine collection for 24 h would be reduced for the subsequent chemotherapy cycles. However, renal function should be evaluated before each chemotherapy cycle, because the change in residual renal function may also affect the AUC of CBDCA. Moreover, 24-h creatinine clearance may be a suitable method to evaluate residual renal function for hemodialysis patients with available daily urine volume.

A previous prospective observational study in patients who received chemotherapy, including CBDCA-based regimens, evaluated the impact of chemotherapy-induced neutropenia on QOL [14]. The authors concluded that there may be a QOL decrement associated with development of grade 4 neutropenia. Thus, the adjustment of treatment intensity is needed to avoid decrement in QOL induced by the development of severe hematologic toxicities. Moreover, it is especially important for elderly patients, because older age is commonly recognized as a risk factor for chemotherapy-induced neutropenia and associated complications [15]. In our elderly patient, adverse events in the second and third cycles could be controlled and stable disease was achieved for 3 cycles by the adjustment of CBDCA dose and hemodialysis timing according to the monitoring the measured AUC. Consequently these 2 cycles of chemotherapy did not decrease the patient’s QOL.

## Conclusions

CBDCA-based chemotherapy is clinically acceptable and can be considered as a treatment option for hemodialysis patients > 80 years-old. However, in such patients, it is essential to measure the AUC of CBDCA and to monitor the severity of hematologic toxicities during chemotherapy, because the measured AUC in the first cycle may unexpectedly exceed the target AUC. Moreover, the AUC is hard to predict due to various influencing factors regarding hemodialysis that may be different for each patient. Therefore, further case reports and prospective studies will be needed to assess the optimal dose and administration procedure for CBDCA.

## Abbreviations

AUC: Area under the concentration–time curve
BSA: Body surface area
CBDCA: Carboplatin
GEM: Gemcitabine
GFR: Glomerular filtration rate
NBS: Norm-based score
QOL: Quality of life
UC: Urothelial cell carcinoma
